# Increase in IL-21 producing T-cells in patients with systemic lupus erythematosus

**DOI:** 10.1186/ar3474

**Published:** 2011-09-29

**Authors:** Sebastian Dolff, Wayel H Abdulahad, Johanna Westra, Berber Doornbos-van der Meer, Pieter C Limburg, Cees GM Kallenberg, Marc Bijl

**Affiliations:** 1 Department of Rheumatology and Clinical Immunology, University Medical Center Groningen, University of Groningen, Hanzeplein 1, 9700 RB Groningen, The Netherlands; 2 Department of Nephrology, University Hospital Essen, University Duisburg-Essen, Hufelandstraße 55, 45122 Essen, Germany

**Keywords:** SLE, Th17-cells, IL-21, T-cells

## Abstract

**Introduction:**

Systemic lupus erythematosus (SLE) is an autoimmune disease accompanied by a disturbed T-cell balance skewed towards effector T-cells, in particular Th17-cells. The novel cytokine interleukin-21 (IL-21) is suggested to be crucial for triggering T-cell responses towards IL-17 producing cells. Thus, we aimed to investigate the ability of T-cells to produce IL-21 and IL-17 in SLE patients.

**Methods:**

Peripheral blood of 34 SLE patients and 18 healthy controls (HC) was stimulated with phorbol myristate acetate (PMA) and calcium ionophore (Ca-Io). Percentages of IL-21- and IL-17A expressing T-cells were analysed by flow cytometry. The expression levels of the transcription factors B-cell lymphoma-6 (BCL-6) and factors retinoid-related orphan receptor (ROR-γt) were assessed in T-cells by real-time RT-PCR and flow cytometry. Additionally, IL-21 receptor (IL-21R) expression on B- and T-cells of patients and HC was analyzed.

**Results:**

Significantly increased percentages of IL-21 expressing CD4^+ ^T-cells and CD8^+ ^T-cells were found in SLE patients as compared to HC. The percentages of IL-21^+ ^CD4^+ ^T-cells and CD8^+ ^T-cells correlated significantly with the percentages of IL-17A^+ ^CD4^+ ^T-cells and CD8^+ ^T-cells, respectively. The relative expression of BCL-6 and ROR-γt did not differ between SLE patients and HC. IL-21R expression occurred mainly on B-cells and was not different comparing SLE patients and HC.

**Conclusions:**

This study demonstrates an increased proportion of IL-21^+ ^T-cells in SLE patients correlating with the proportion of IL-17^+ ^T-cells. This suggests a pivotal role of IL-21 in the pathogenesis of SLE.

## Introduction

Systemic lupus erythematosus (SLE) is an autoimmune disease of unknown etiology. The presence of antibodies against dsDNA is a hallmark of SLE. Although the precise pathogenesis of SLE has not been fully elucidated, disturbances in T-cell and B-cell homeostasis appear to contribute to the inflammatory pathology of SLE. Several cytokines have been demonstrated to be crucial for the regulation of B- and T-cell homeostasis. Recently, the novel cytokine interleukin (IL)-21 has been found to play a pivotal role in differentiation and function of T-cells. In particular, IL-21 drives an inflammatory T-cell response by triggering the production of IL-17, which is thought to be a crucial cytokine for inflammatory processes as occur in lupus nephritis in SLE [1].

The novel class I cytokine IL-21 is a member of the common γ-chain receptor family. The production of IL-21 is mainly restricted to CD4^+ ^T-cells, Th17- and T-follicular helper (T_FH_)-cells. In addition, natural killer (NK) T cells have been demonstrated to be potent IL-21 porducers *in vitro *[2]. High expression of the transcription factors retinoid-related orphan receptor (ROR)-γt and B-cell lymphoma-6 (BCL6) in T-cells is considered to define specifically Th17- and T_FH _-cell lineages, respectively. The unique IL-21 receptor (IL-21Rα) can be expressed on various cell types, including T- and B-cells, NK cells, dendritic cells and macrophages [3,4]. Ligation of IL-21 to its receptor IL-21Rα promotes B-cell dependent IgG production, enhances expansion of CD8^+ ^cells and their cytotoxic capacity, and augments naïve CD4^+ ^T-cell differentiation towards effector T-cells [1,5,6].

Multiple murine models indicate a pivotal role of IL-21 in the pathogenesis of autoimmune diseases [7-9]. In an animal model of rheumatoid arthritis, blocking the IL-21 pathway by administration of a fusion protein (IL-21R.Fc) ameliorated disease severity [9]. In addition, Fina *et al*. reported high levels of IL-21 in the inflamed colon of wild-type mice, which developed colitis after treatment with dextran sulfate sodium (DSS) as a model of inflammatory bowel disease [8]. Further, they demonstrated that IL-21 knockout mice were protected against DSS induced inflammatory bowel disease [8]. The authors suggested a reduced Th17 response in IL-21 deficient mice as an underlying mechanism which might be beneficial in this murine model. Using a BXSB-*Yaa *SLE murine model, it was demonstrated that mice which are IL-21R-deficient show less lupus-like symptoms as compared to wild type BXSB-*Yaa *mice [7].

Human studies provide further evidence that the IL-21/IL-21R pathway plays a major role in the pathogenesis of autoimmune diseases, in particular in SLE. Plasma levels of IL-21 were significantly elevated in SLE patients in comparison with healthy controls [10]. Sawalha *et al*. reported an association of two SNPs (single nucleotide polymorphisms) located in the IL-21 gene with SLE but functional data were not provided in this study [11]. In general, functional data on IL-21 expression in human SLE and their potential link to IL-17A producing effector T-cells are lacking so far.

Therefore, we aimed to elucidate the role of IL-21 in the context of Th17-cells in the pathogenesis of human SLE. We hypothesized that increased IL-21 production is present in SLE patients. This might be correlated with the Th17 response in SLE patients. In addition, the study aimed to clarify whether Th17 cells are a source of IL-21 in SLE patients. To investigate this, peripheral whole blood was stimulated and the percentages of IL-21 positive and Th17 positive T-cells were analysed. Moreover, the expression of the transcription factors of T_FH_- and Th17-cell specific messenger RNAs (mRNAs), BCL6 and ROR-γt, respectively, was determined in isolated unstimulated T-cells and stimulated T-cells. To test whether B- and T-cells are susceptible for IL-21 signalling we analysed the proportion of IL-21R expressing B- and T-cells in HC and SLE patients.

## Materials and methods

### Study population

Consecutive SLE patients (*n *= 34) aged 41 ± 15 mean (± SD) years attending the outpatient clinic and 18 age- and sex-matched healthy controls (age 39 ± 12 years) were enrolled in this study. All patients fulfilled at least four of the American College of Rheumatology's revised criteria for SLE [12]. Disease activity was assessed by the SLEDAI (SLE Disease Activity Index). Twenty-seven patients had inactive disease (SLEDAI score ≤ 4) and seven patients had active SLE (defined as SLEDAI score > 4). Median disease activity for all patients was 4 (range 0 to 17). Four patients did not receive any immuno-modulating medication at the time of analysis (Table 1). Informed consent was obtained from patients after approval of the study by the Medical Ethics Committee of the University Medical Center Groningen. The study was conducted according to the ethical guidelines of our institution and the Declaration of Helsinki.

**Table 1:** Baseline characteristics and medication of SLE patients (*n *= 34) and healthy controls (HC, *n *= 18) included in the study.

	*SLE patients*	*HC*	*P-value*
Total number	34	18	
Women/men	28/6	15/3	ns
Age (years, mean ± SD)	41 ± 14	35 ± 11	ns
SLEDAI (median (range))	4 (0 to 17)		
C3, g/l (median, range)	0.82 (0.37 to 1.45)		
C4, g/l (median, range)	0.21 (0.04 to 0.30)		
Anti-dsDNA, E/ml (median, range)	165 (4 to 1,000)		
Treatment, n		18	
None	4		
Glucocorticoids, n	23		
*median dose (range), dose (mg/day)*	5 (3.75 to 60)		
Immunosupressive/immunmodulating, n			
Hydroxychloroquine	17		
*median dose (range), users (mg/day)*	400 (200 to 600)		
Methotrexate	2		
*median dose (range), users (mg/week)*	15 (5 to 25)		
Azathioprine	12		
*median dose (range), users (mg/day)*	112.5 (50 to 150)		
MMF	4		
*median dose (range), users (mg/day)*	2,500 (1,000 to 3,000)		

SLEDAI: systemic lupus erythematosus disease activity index, MMF: mycophenolate mofetil.

### Stimulation assay and immunofluorescent intracellular staining for cytokines

Sodium heparinized venous blood was obtained from all participants. Immediately after sampling, 200 μl blood was mixed with 200 μl RPMI1640 (Cambrex Bio Science, Verviers, Belgium), supplemented with 50 μg/ml gentamycin (Gibco, Paisley, Scotland, UK), and aliquoted into 5 ml polypropylene tubes (BD Biosciences, Amsterdam, The Netherlands)) (400 μl per tube). To determine the frequency of cytokine expressing T-cell subsets, diluted blood was stimulated for 4 h with 40 nM phorbol myristate acetate (PMA; Sigma-Aldrich, Steinheim, Germany) and 2 nM calcium ionophore (Ca-Io; Sigma-Aldrich) in the presence of 3 μM Brefeldine A. Brefeldine A was used to block intracellular transport mechanisms, thereby leading to an accumulation of cytokines in the cell. As a negative control, one sample remained without stimulation. Culture tubes were incubated at 37°C, 5% CO_2_.

After stimulation, cells were incubated with 2.5 ml amoniumchloride (pH 7.4) on ice. Lysed erythrocytes were washed in wash buffer (PBS, 5% fetal bovine serum (FBS), 0.1% sodium azide (Merck, Darmstadt, Germany)) and stained with PerCP- conjugated anti-CD8 (clone SK1, BD Biosciences, Amsterdam, The Netherlands) and allophycocyanin (APC)-conjugated anti-CD3 (clone UCHT1, BD Biosciences), for 15 minutes at room temperature. Cells were fixed with 100 μl Reagent A (Caltag Laboratories, An der Grab, Austria) for 10 minutes. After washing, the pellet was resuspended in 100 μl permeabilization Reagent B (Caltag Laboratories) and labeled with Alexa Fluor 488-conjugated anti-IL17A (clone eBio64Dec17) and PE-conjugated anti-IL21 (clone ebio3A3-N2), (both purchased from eBioscience, Uithoorn, The Netehrlands)), for 20 minutes in the dark. After staining, the cells were washed and immediately analyzed on FACS-Calibur flow cytometer (BD Biosciences).

### Stimulation assay and immunofluorescent staining for transcription factors

Peripheral blood mononuclear cells (PBMCs) from patients and matched healthy controls were prepared from heparinized venous blood by density-gradient centrifugation on Lymphoprep (Axis-Shield PoC AS, Oslo, Norway) immediately after blood was drawn. Cells recovered from the gradient interface were washed twice in PBS, adjusted to 1 × 10^7 ^cells/ml, and stimulated for 4 h with PMA and Ca-Io as aforementioned. After stimulation, staining for BCL6 and ROR-γt was performed according to the manufacturer's instructions (eBioscience staining set for transcription factors). Briefly, stimulated and unstimulated PBMCs were adjusted to 1 × 10^7 ^cells in 100 μl and incubated with an appropriate concentration of eFluor450- conjugated anti-CD3 (clone OKT3, eBioscience), and PerCP-conjugated anti-CD8 (clone SK1, BD Biosciences) for 30 minutes at 4°C in the dark, followed by fixation and permeabilizaion in Fix/Perm buffer (eBioscience) for 45 minutes. Cells were then washed twice with 1 × permeabilization buffer (eBioscience), and stained with PE-conjugated anti-BCL6 (clone 603406, R&D Systems, Abingdon, UK)) and APC-conjugated anti-ROR-γt (cloneAFKJS-9, eBioscience). After incubation for 30 minutes in the dark, the cell suspension was washed and four-color staining was immediately analyzed on FACS-LSRII flow cytometer (BD Biosciences).

### Immunofluorescent surface staining for IL-21R on B- and T- cells

Blood samples were labeled with the following monoclonal antibodies: PE-conjugated anti-IL21R, APC-conjugated anti-CD3, PerCP-conjugated anti-CD4, FITC-conjugated anti-CD19 (BD Biosciences, Amsterdam, The Netherlands) for 15 minutes in the dark. Afterwards, cells were successively treated with 2 ml diluted FACS lysing solution (BD Biosciences) for 10 minutes and then washed twice in wash-buffer and immediately analyzed by flow cytometry. Four-color staining was analyzed on FACS-Calibur (BD Biosciences).

### Flow cytometric analysis

**Figure 1:**
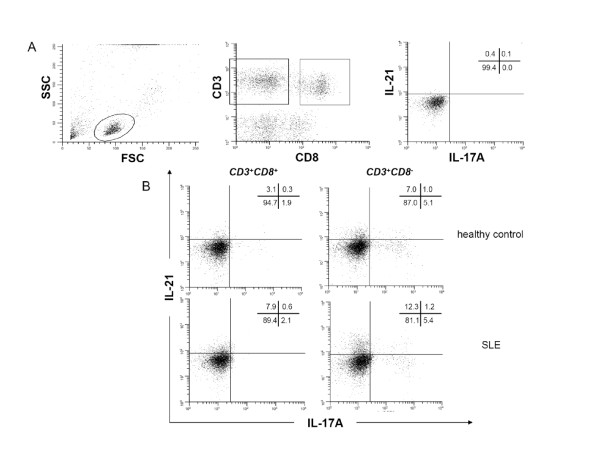
**Increased IL-21 expression in T-cells of SLE patients**. This figure shows a representative dot plot of cytokine expression in unstimulated (**A**) and stimulated (**B**) blood samples. Due to the down-regulation of CD4 after stimulation with PMA/Ca-Io cells were gated for CD3^+^CD8^+ ^and CD3^+^CD8^- ^T-cell subsets. Unstimulated samples were used as negative controls. The expression of IL-17A and IL-21 is shown for a healthy control and an SLE patient.

Four-color flow cytometric acquisition was performed using Cell Quest software (Becton-Dickinson). For all flow cytometric analyses, data were collected for 4 × 10^5 ^cells, and plotted using the Win-List software package (Verity Software House Inc., Topsham, ME, USA). Because stimulation reduces surface expression of CD4 on T-cells, CD4^+ ^T-cells were identified indirectly by gating on CD3-positive and CD8-negative lymphocytes. The unstimulated samples were used as a guide for setting the linear gates to delineate positive and negative populations for the cytokine production (Figure 1), whereas appropriate isotype-matched controls were used for setting the gates to quantify the percentage of T-cells expressing a specific transcription factor.

### Cell isolation and sorting of CD4^+ ^T-cells

Isolated PBMCs were frozen in RPMI 1640 (Cambrex Bioscience, Verviers, Belgium) supplemented with 10% fetal calf serum (FCS), 50 μg/ml of gentamicin (Gibco, Paisley, UK) and 10% dimethylsulfoxide. PBMCs were stored in liquid nitrogen. PMBCs were thawed gently in 10% FCS supplemented RPMI 1640 medium at the day of sorting. After washing, the cells were stained with PerCP-conjugated anti-CD8 (clone SK1, BD Biosciences) and allophycocyanin (APC)-conjugated anti-CD3 (clone UCHT1, BD Biosciences), for 15 minutes at room temperature and washed again. Sorting of CD4^+ ^T-cells was performed by gating the CD3^+^CD8^- ^cell population.

### RNA isolation and real-time RT-PCR

RNA was isolated from CD4^+ ^cells with TRIzol reagent (Invitrogen, Bleiswijk, The Netherlands) according to the manufacturer's instructions. DNAse treatment (Ambion, Huntingdon, Cambridgeshire, UK) was performed and subsequently cDNA was synthesized using M-MLV reverse transcriptase and oligo (dT) 14 to 18. For measurement of mRNA for BCL6 and ROR-γt and glyceraldehyde-3-phosphate dehydrogenase (GAPDH), 1 μl of cDNA in triplicate was used for amplification by the Taqman RT-PCR system (ABI Prism 7900HT Sequence Detection System, Applied Biosystems, Foster City, CA, USA) with specific Taqman primers/probes (Applied Biosystems). Amplification was performed using standard conditions and calculations of fold induction were performed. We normalized gene expression to GAPDH and expressed values relative to control using the ^ΔΔ^CT method.

### Statistical analysis

Data are presented as mean ± SD unless stated otherwise. The nonparametric Mann-Whitney U-test was used to compare data between SLE patients and healthy controls, and differences were considered statistically significant at two-sided *P*-values less than 0.05. Paired samples were analysed by Wilcoxon matched paired test. Correlation analysis was performed using Spearman's rank correlation coefficient.

## Results

### Increased percentages of IL-21^+ ^T-cells in SLE patients

**Figure 2:**
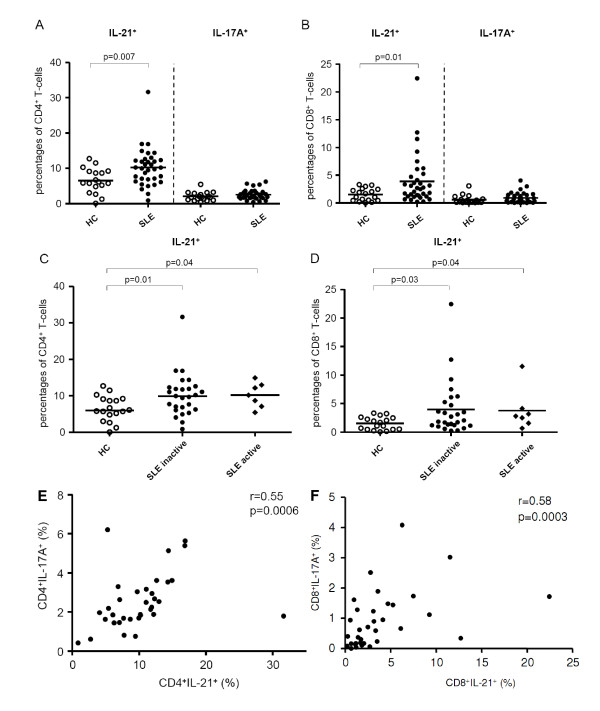
**Multiparameter flow cytometric analysis of PMA/Ca-Io induced cytokine expression in CD4^+ ^T-cells and CD8^+ ^T-cells**. Whole blood from SLE patients and healthy controls (HC) was stimulated with PMA/Ca-Io and analyzed for intracellular cytokine expression of IL-21 and IL-17A among CD4^+ ^T-cells and CD8^+ ^T-cells (**A-F**). Frequencies of these T-cells are shown. Horizontal lines represent the mean. *P*-values were calculated using the nonparametric Mann-Whitney U-test.

In order to detect intracellular cytokine expression whole blood was stimulated. Unstimulated T-cells of SLE patients and HC did not spontaneously express IL-21 or IL-17 (data not shown). Intracellular cytokine expression was analysed after *in vitro *activation of CD4^+ ^T-cells and CD8^+ ^T-cells. Significantly increased percentages of IL-21 expressing CD4^+ ^T-cells were found in SLE patients as compared to HC (10.2 ± 5.4% vs. 6.5 ± 3.5%, *P *= 0.007). Percentages of IL-21 expressing CD8^+ ^T-cells were also significantly increased in SLE patients as compared to HC (3.9 ± 4.5% vs. 1.5 ± 1.1%, *P *= 0.01, Figure 2A, B).

### Percentages of IL-21 ^+ ^T-cells in active and inactive SLE patients

To determine whether the intracellular IL-21 expression was associated with disease activity we analyzed the proportion of IL-21 producing T-cells in HC, active and inactive SLE patients, respectively (Figure 2C). The percentages of IL-21 expressing cells within the CD4^+ ^T-cell population were increased in inactive patients as compared to HC (10.2 ± 5.8% vs. 6.5 ± 3.4%, *P *= 0.01). Mean percentages of IL-21 expressing CD4^+ ^T-cells in active patients were also significantly increased as compared to HC (10.2 ± 3.4% vs. 6.5 ± 3.4%, *P *= 0.04), but not higher than in inactive patients.

Percentages of IL-21 expressing cells within the CD8^+ ^T-cells were also increased in inactive patients as compared to HC (4.0 ± 4.8% vs. 1.5 ± 1.2%, *P *= 0.03). There was an increased proportion of IL-21 expressing CD8^+ ^T-cells in active SLE patients as compared to HC (3.8 ± 3.6% vs. 1.5 ± 1.1%, *P *= 0.04, Figure 2D). Additionally, there was no correlation between the percentages of IL-21 expressing cells within the CD4^+ ^or CD8^+ ^T-cell population and SLE disease activity index (r = 0.15, *P *= 0.39 and r = 0.14, *P *= 0.45, respectively). Also, no correlation was present between percentages of IL-21 expressing cells within the CD4^+ ^or CD8^+ ^T-cell population and complement levels (C3 and C4) or anti-dsDNA autoantibody-titres (data not shown).

In order to assess the influence of treatment on the proportion of IL-21 expressing CD4^+ ^T-cells we performed a subanalysis comparing patients without medication or on prednisolone only (*n *= 8) with patients on a combination of immunosuppressives (*n *= 26). There was no significant difference in the proportion of IL-21 expressing CD4^+^- and CD8^+ ^T-cells between these groups (8.2 ± 4.6% vs. 10.4 ± 5.7%, n.s. and 3.1 ± 2.9% vs. 4.2 ± 4.9% n.s.).

### Percentages of IL-21^+ ^T-cells correlate with percentages of IL-17A^+ ^T-cells

In order to analyze the relation between IL-21^+ ^T-cells and IL-17A^+ ^T-cells, we correlated percentages of IL-21^+ ^T-cells with percentages of IL-17A^+ ^T-cells in SLE patients and HC. Interestingly, there was a significant correlation between percentages of CD4^+^IL-21^+ ^-T-cells and CD4^+^IL-17A^+ ^T-cells in SLE patients (r = 0.55, *P *= 0.0006) (Figure 2E). Also, a significant correlation was found between percentages of CD8^+^IL-21^+ ^-T-cells and CD8^+^IL-17A^+ ^T-cells in SLE patients (r = 0.5, *P *= 0.0003) (Figure 2F).

### Proportions of IL-21R expressing cells are highest within CD19^+ ^B-cells in SLE patients and HC

**Figure 3:**
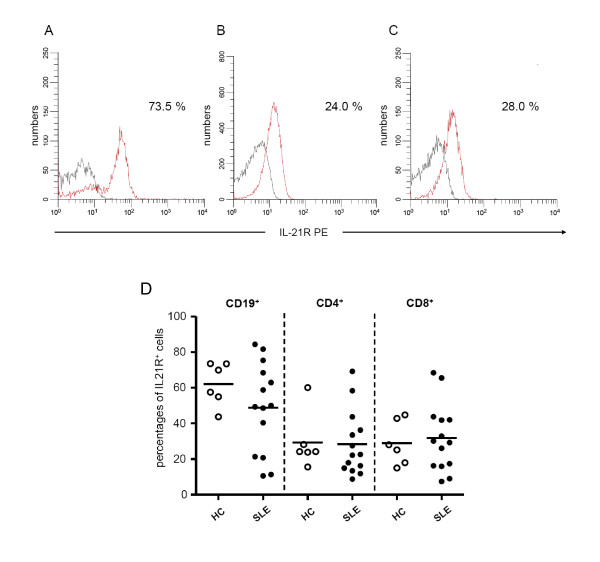
**Expression of IL-21R on B- and T-cells of SLE patients**. The histograms show a representative example of the expression of IL-21R on CD19^+^-cells (**A**), CD4^+^-cells (**B**) and CD8^+^-cells (**C**). Positive staining (red line) was compared to an isotype control (grey line). The proportion of receptor positive cells is indicated. **D **shows the IL-21R expression on CD19^+ ^B-cells and CD4^+ ^and CD8^+ ^T-cells from SLE patients (*n *= 14) in comparison with healthy controls (HC, *n *= 6). Horizontal lines represent the mean. *P*-values were calculated using the nonparametric Mann-Whitney U-test.

An altered IL-21/IL21R signalling might also be explained by different levels of IL-21R expression. Therefore, the expression of IL-21R on B- and T-cells was analysed as well (Figure 3). There was no difference in IL-21R expression on CD19^+^, CD4^+ ^and CD8^+ ^cells between SLE patients (*n *= 14) and HC (*n *= 6). The highest proportions of IL-21R expressing cells were found in the CD19^+ ^B-cell subset. In SLE patients, 48.9 ± 25.0% of CD19^+ ^B-cells were expressing the IL-21R vs. 62.2 ± 12.0% in HC (*P *= 0.34). Percentages of CD4^+ ^T-cells expressing the IL-21R were 28.3 ± 18.1% in SLE patients as compared to 29.4 ± 15.6% in HC (*P *= 0.53). Percentages of CD8^+ ^T-cells expressing the IL-21R were 31.9 ± 19.0% in SLE patients as compared to 29.0 ± 12.5% in HC (*P *= 0.53).

### Relative expression of BCL6 and ROR-γt is not altered in SLE

**Figure 4:**
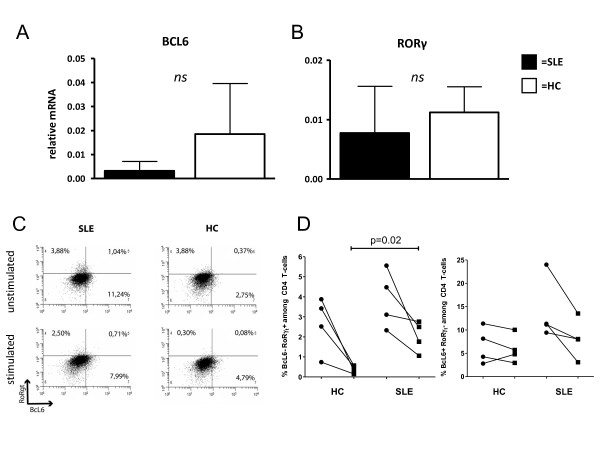
**Relative expression of transcription factors BCL6 and ROR-y**. The relative mRNA expression of BCL6 and ROR-y in unstimulated, sorted CD4^+ ^T-cells was determined in SLE patients (SLE, *n *= 7) and healthy controls (HC, *n *= 4) (**A/B**). There was no significant difference in the relative expression between these two groups. Bars represent the mean values ± SD. In (**C**) a representative dot plot of unstimulated and stimulated PBMCs from a SLE patient a healthy control is shown. The percentages of BCL6 and ROR- γt^+ ^CD4^+ ^T-cells in unstimulated (dots) and stimulated samples (squares) are given (**D**).

To test whether T_FH_- and Th17 specific transcription factor expression is altered in SLE, RNA was isolated from sorted CD4^+ ^T-cells of SLE patients (*n *= 7) and HC (*n *= 4) (Figure 4A, B). The relative expression of CD2 mRNA served as a positive control. The expression levels were not different between SLE patients and HC (0.337 ± 0.152 vs. 0.267 ± 0.066). The relative expression of BCL6 was 0.003 ± 0.004 in CD4^+ ^T-cells of SLE patients as compared to 0.02 ± 0.02 in HC (*P *= 0.07). The relative expression of ROR-γt was low and also not significantly different between SLE patients and HC (0.007 ± 0.007 vs. 0.01 ± 0.004, *P *= 0.3).

### Percentages of BCL6^+ ^and ROR-γt^+ ^CD4^+ ^T-cells do not differ between unstimulated and stimulated conditions

Next, the expression of the transcription factors BCL6 and ROR-γt were assessed in CD4^+ ^T-cells, in both unstimulated and stimulated samples, by flow cytometry. The percentages of ROR-γt^+ ^in unstimulated CD4^+ ^T-cells of SLE patients (*n *= 4) were 3.8 ± 1.4% vs. 2.0 ± 0.8% (n.s.) (Figure 4). There was also no significant difference in the proportion of BCL6^+ ^CD4^+ ^T-cells of SLE patients after stimulation with PMA and Ca-Io (14.0 ± 6.7% vs. 8.2 ± 4.3%, n.s.). Furthermore, the analysis of healthy controls (*n *= 4) revealed no significant difference of the percentages of ROR-γt^+ ^CD4^+ ^T-cells or BCL6^+ ^CD4^+ ^T-cells after stimulation, respectively. (ROR-γt^+^: 2.6 ± 1.4% vs. 0.4 ± 0.2% and BCL6^+^: 6.6 ± 3.9% vs. 5.9 ± 3.0%). There was also no significant difference between the percentages of BCL6^+ ^and ROR-γt^+ ^cells among the CD8^+ ^T-cells in SLE patients and healthy controls (data not shown).

## Discussion

The present study is the first demonstrating increased proportions of IL-21^+ ^T-cells in SLE patients in comparison with HC. Increased proportions of IL-21^+ ^cells could be observed in both CD4^+ ^and CD8^+ ^T-cells.

The IL-21/IL-21R pathway seems to play an important role in the homeostasis of T-cells, in particular in the differentiation of naïve T-cells towards Th17 cells. These cells have been identified to be key mediators in autoimmune diseases such as SLE [13-15]. Additionally, Th17 cells have been shown to be a source of IL-21 [16]. It is likely that IL-21 induces CD4^+ ^T-cells to produce IL-17 and that IL-17 amplifies the effector response via an autocrine loop [17]. To elucidate whether Th17 cells in SLE are a source of IL-21 we analyzed the proportion of IL-21^+ ^T-cells in combination with IL-17^+ ^T-cells. Almost no IL-21^+ ^IL-17^+ ^double positive cells were detectable in SLE patients and HC upon stimulation with PMA/Ca-Io. This indicates that mainly other T-cells, such as T-follicular helper cells (T_FH_), are the source of this cytokine [18]. Although the relative expression of BCL6, the master regulator of T_FH_, was not increased in unstimulated T-cells of SLE patients in comparison with HC, other studies suggest that T_FH _are the main source of IL-21 [19]. A study by Simpson *et al*. has shown that BCL6 expression is relatively low in all peripheral T-cell subsets in comparison to tonsillar T_FH _[20]. Remarkably, in mice the number of circulating T_FH _is independent of circulating serum IL-21. This might be different in humans. In the present study, we showed that the expression level of BCL6 in sorted T-cells from peripheral blood of SLE patients and HCs was relatively low. This is in line with the findings of Simpson *et al*. Remarkably, the proportion of BCL6 expression in CD4^+ ^T cells, analyzed by flow cytometry method, tended to be higher in SLE patients; however, this did not reach significance. In addition, the proportion of BCL6^+^CD4^+ ^T-cells and ROR-γt^+^CD4^+ ^T-cells in unstimulated samples were in agreement with those of the *in vitro *induced cytokine expression of IL-21 and IL-17, respectively.

We found a strong positive correlation between percentages of IL-21^+ ^CD4^+ ^T-cells and percentages of IL-17^+ ^CD4^+ ^T-cells in SLE patients. The same correlation was found for CD8^+ ^T-cells. In line with these results a similar correlation between IL-21^+ ^CD4^+ ^T-cells and Th17 cells has been reported in patients with autoimmune thrombocytopenia (ITP) [21]. However, the proportion of IL-17 producing T-cells was not significantly increased in SLE patients compared to HC. This was probably due to the low numbers of patients included. Furthermore, recently it has been shown that IL-21 in the presence of IL-6, (which is increased in part of the SLE patients as well), promotes differentiation towards Tfh cells instead of towards Th-17 cells [22,23].

Subanalysis of the proportions of IL-21^+ ^T-cells revealed no difference between active and inactive disease. Moreover, there was no correlation between proportions of IL-21^+ ^T-cells and the SLE Disease Activity Index (SLEDAI). Although this could be due to the relatively small group of active patients who were enrolled, an intrinsic abnormality is possible as well. We did not find an influence of treatment on the proportion of IL-21^+ ^T-cells in SLE.

T- and B-cells have been reported to be targets of IL-21. IL-21 influences immunoglobulin production and promotes B-cell expansion and plasma cell generation [5,24]. T-cell dependent autoantibody production might also be regulated by IL-21. The comparable expression of IL-21R on T-cells between SLE patients and HC suggests that patients have the same abilitiy to respond to IL-21 as HC. The high percentage of B-cells expressing IL-21R in the present study encourages the idea that the IL-21/IL-21R pathway might be important for B-cell function. A study by Mitoma *et al*. reported that mainly naive B-cells express the IL21R but also memory B-cells and plasmablasts [25]. In contrast to our results, the authors found significantly lower levels in SLE patients in comparsion with HC. First, this might be explained by differences in staining procedures. Secondly, differences in clinical characteristics might have influenced the results, but clinical characteristics were not described in the paper by Mitoma *et al*. [25].

Additionally, increased IL-21 plasma levels have been found in SLE patients [10]. Therefore, we tested whether there was a correlation between percentages of IL-21^+^CD4^+ ^T-cells and anti-dsDNA titres. However, there was no correlation between these variables. This is likely due to the fact that anti-dsDNA production *in vivo *is complex and dependent on co-stimulation, various cytokines and soluble factors. The percentages of circulating plasma cells as a source of anti-dsDNA antibodies might be associated with titres but were not analysed.

IL-21^+ ^seems to regulate the suppressive capacity of T-cells through the inhibition of T_reg _function and on the other hand to amplify effector T-cells by promoting the Th17 response [8,26]. In the present study, we demonstrate an increased proportion of IL-21^+ ^T-cells, which correlate with the proportion of IL-17^+ ^T-cells in SLE. Thus, IL-21^+ ^T-cells might contribute to the generation of pathogenic T-cells in this autoimmune disease. Blocking the IL-21/IL-21R pathway by administration of a fusion protein has been fruitful in animal models of RA and in a lupus-prone MRL-*Fas^lpr ^*mouse model [8,27]. Treatment with IL-21R.Fc reduced renal disease, skin lesions and circulating auto-antibodies in these MRL-*Fas^lpr ^*mice. Remarkably, CD4^+ ^and CD8^+ ^T-cells in the spleen of these mice were reduced after treatment with IL-21R.Fc supporting the idea that IL-21 has also a major impact on T-cell populations.

## Conclusions

In conclusion, this study demonstrates increased proportions of IL-21^+ ^T-cells in SLE patients, which were correlated with proportions of IL-17^+ ^T-cells. Inhibiting the IL-21/IL-21R pathway has been shown to be effective in ameliorating disease severity in lupus mouse models. Targeting IL-21 in human SLE could be a promising approach in the future [28]. Therefore, further investigations are necessary to confirm and extend the current results.

## Abbreviations

APC: Allophycocyanin; BCL6: B-cell lymphoma-6; Ca-Io: calcium ionophore; DSS: dextran sulfate sodium; FBS: fetal bovine serum; FCS: fetal calf serum; FITC: fluorescein isothiocyanate; GAPDH: glyceraldehyde-3-phosphate dehydrogenase; HC: healthy controls; IL: interleukin; ITP: autoimmune thrombocytopenia; NK: natural killer; PBMCs: peripheral blood mononuclear cells; PBS: phosphate buffered saline; PE: Phycoerythrin; PerCP: peridin chlorophyll protein; PMA: phorbol myristate acetate; (ROR)-γt: factors retinoid-related orphan receptor; SLE: systemic lupus erythematosus; SLEDAI: Systemic Lupus Erythematosus Disease Activity Index; SNPs: single nucleotide polymorphisms.

## Competing interests

The authors declare that they have no competing interests.

## Authors' contributions

All authors contributed to the design, and acquisition and interpretation of data. SD and WHA performed the flowcytometry and *in vitro *experiments and drafted the manuscript. SD performed the statistical analysis and contributed to the interpretation of the data. JW and BDvdM contributed to the aquisition and interpretation of RT-PCR experiments. PCL contributed to concept and design, and interpretation of data. CGMK contributed to concept and design, interpretation of data and revising the manuscript. MB contributed to concept and design, inclusion of SLE patients, interpretation of data and drafting of the manuscript.
